# The DNA relaxation-dependent OFF-to-ON biasing of the type 1 fimbrial genetic switch requires the Fis nucleoid-associated protein

**DOI:** 10.1099/mic.0.001283

**Published:** 2023-01-12

**Authors:** Colin Conway, Michael C. Beckett, Charles J. Dorman

**Affiliations:** ^1^​ Department of Microbiology, Moyne Institute of Preventive Medicine, Trinity College Dublin, Dublin 2, Ireland; ^†^​ Present address: Technical University of the Atlantic, Galway, Ireland

**Keywords:** DNA supercoiling, Fis, nucleoid-associated protein, nucleoprotein complexes, type 1 fimbriae

## Abstract

The structural genes expressing type 1 fimbriae in *Escherichia coli* alternate between expressed (phase ON) and non-expressed (phase OFF) states due to inversion of the 314 bp *fimS* genetic switch. The FimB tyrosine integrase inverts *fimS* by site-specific recombination, alternately connecting and disconnecting the *fim* operon*,* encoding the fimbrial subunit protein and its associated secretion and adhesin factors, to and from its transcriptional promoter within *fimS*. Site-specific recombination by the FimB recombinase becomes biased towards phase ON as DNA supercoiling is relaxed, a condition that occurs when bacteria approach the stationary phase of the growth cycle. This effect can be mimicked in exponential phase cultures by inhibiting the negative DNA supercoiling activity of DNA gyrase. We report that this bias towards phase ON depends on the presence of the Fis nucleoid-associated protein. We mapped the Fis binding to a site within the invertible *fimS* switch by DNase I footprinting. Disruption of this binding site by base substitution mutagenesis abolishes both Fis binding and the ability of the mutated switch to sustain its phase ON bias when DNA is relaxed, even in bacteria that produce the Fis protein. In addition, the Fis binding site overlaps one of the sites used by the Lrp protein, a known directionality determinant of *fimS* inversion that also contributes to phase ON bias. The Fis–Lrp relationship at *fimS* is reminiscent of that between Fis and Xis when promoting DNA relaxation-dependent excision of bacteriophage λ from the *E. coli* chromosome. However, unlike the co-binding mechanism used by Fis and Xis at λ *attR*, the Fis–Lrp relationship at *fimS* involves competitive binding. We discuss these findings in the context of the link between *fimS* inversion biasing and the physiological state of the bacterium.

## Introduction

Type 1 fimbriae (or pili) are surface appendages found on members of the *Enterobacteriaceae* [1]. They are virulence factors in pathogenic strains [2–4] and contribute to biofilm formation in the host [5–7] and in the external environment [8]. Fimbriae are up to 2 µm in length, similar to the length of an *Escherichia coli* cell, and 7 nm in diameter. Each of the fimbriae has a helical structure composed of repeated copies of the FimA subunit protein; the FimH adhesin is located at the tip, where it is responsible for binding to mannose [1]. Mannose-sensitive agglutination of red blood cells is a diagnostic test for the presence of type 1 fimbriae [1, 9–11].

**Fig. 1.**
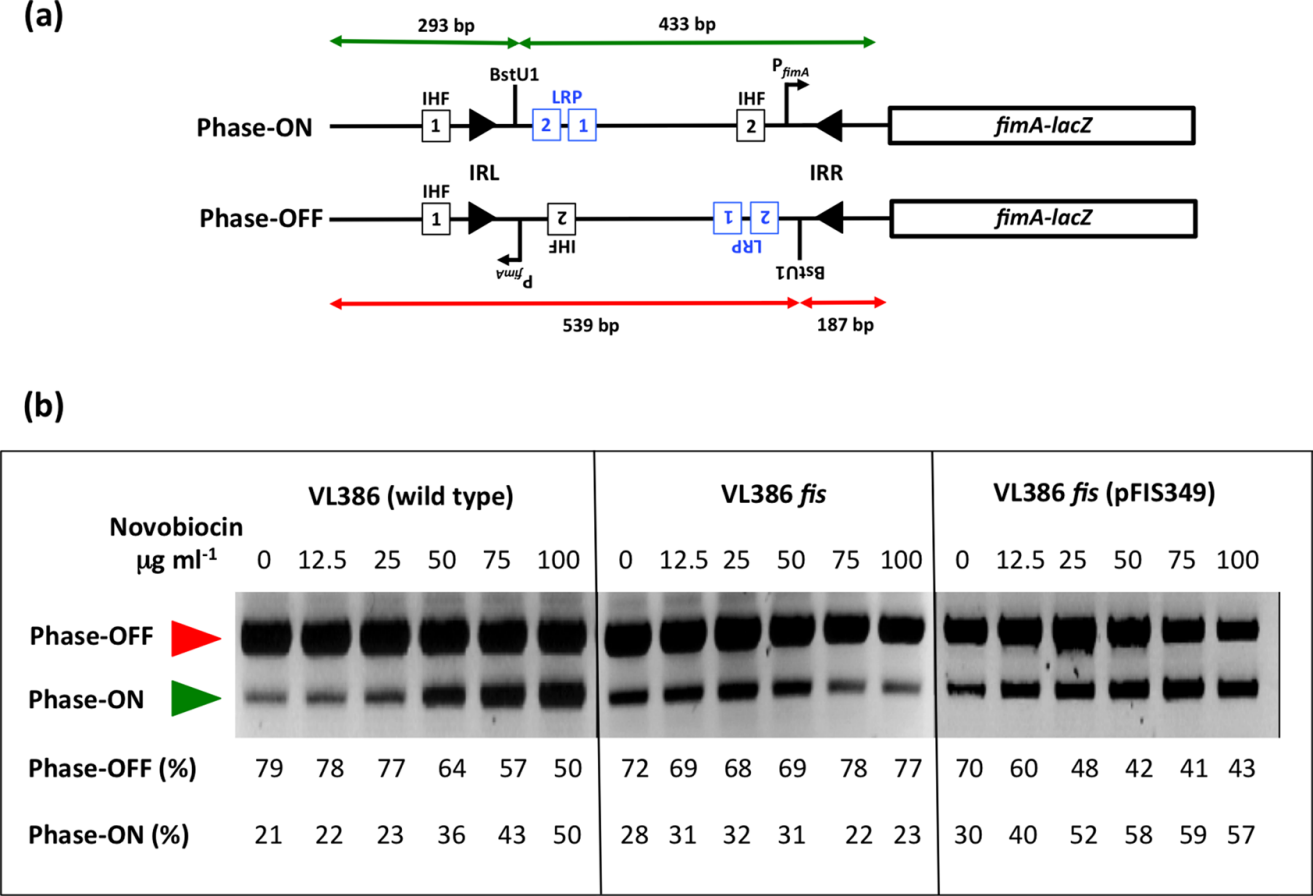
The phase OFF-to-phase ON bias of *fimS* genetic switch inversion in novobiocin-treated cultures is reversed in the absence of the Fis protein. (**a**) The *fimS* inversion assay. The *fimS* genetic element is amplified from the chromosome by PCR and the amplimers are cleaved with the BstUI restriction endonuclease (see Methods). The lengths of the cleavage products are summarized for the ON (green) and OFF (red) orientations of *fimS*, above and below the drawings, respectively. The angled arrow, labelled P*_fimA_*, shows the position and orientation of the transcriptional promoter of the *fimA* gene. In strain VL386 and its derivatives, the *fimA* gene is fused to a *lacZ* reporter gene, allowing phase ON and phase OFF bacterial colonies to be distinguished on MacConkey lactose indicator agar plates. Squares represent the binding sites for IHF (black) and Lrp (blue), respectively. The filled arrowheads represent the left (IRL) and right (IRR) 9 bp inverted repeats that flank *fimS*. The position of the BstUI restriction endonuclease recognition site between Lrp binding site LRP-2 and IRR is shown. Not to scale. (**b**) Electrophoresis of the *fimS* DNA fragments from the wild-type strain (VL386), its *fis* knockout derivative and the complemented *fis* mutant, following BstUI digestion of the PCR-amplified *fimS* genetic element. The red arrowhead indicates the 539 bp phase OFF diagnostic band and the green arrowhead shows the 433 bp diagnostic band. The cultures had been treated with novobiocin at the concentrations given above each gel lane. The intensities of the DNA bands corresponding to the ON and OFF orientations of *fimS* in each lane were determined by densitometry and are reported as percentages below the lane. The experiment was performed three times and typical data are presented.

The production of type 1 fimbriae is subject to phase variation, with fimbriate and afimbriate cells coexisting in the same population [9, 12]. This behaviour has been interpreted as a bet-hedging strategy that balances the risks of producing these highly immunogenic fimbriae (detection by the host immune system; the physiological cost of making, exporting and assembling the structures) with the benefits (biofilm-based community living; colonization of a host or another environmental niche) [13–16]. The invertible *fimS* genetic element is the basis of phase-variable *fim* operon expression. This 314 bp DNA segment harbours both the promoter for the transcription of the *fim* operon (Fig. 1a) and a Rho-dependent transcription terminator that influences the stability of the mRNA transcribed from the *fimE* gene [17, 18]. Inverting *fimS* connects/disconnects the *fim* operon to/from its transcription promoter, and connects/disconnects the *fimE* gene to/from its terminator, affecting FimE production [17].

Inversion of *fimS* involves site-specific recombination within the 9 bp inverted repeats that flank the element [19, 20] (Fig. 1a). In pathogenic strains of *E. coli,* the paralogous, independently acting tyrosine integrases, FimB and FimE, promote inversion [21–24]. These integrases catalyse the recombination reaction using the same chemistry, but their distinct DNA binding preferences at the alternate forms of *fimS* determine their recombination biases: FimB inverts both the phase ON and phase OFF forms of *fimS* with equal efficiency, whereas FimE has a strong preference for the phase ON form, biasing FimE-mediated recombination in favour of ON-to-OFF switching [19, 20, 24, 25]. In bacteria producing both FimB and FimE, the ON-to-OFF inversion preference of FimE predominates and inversion of the *fimS* element is biased strongly towards the OFF orientation [17–19, 25]. Many laboratory strains of *E. coli* K-12 lack the FimE recombinase due to mutations in the *fimE* gene; in these strains, *fimS* inversion depends on the unbiased FimB recombinase alone [26].

FimB-dependent inversion of *fimS* is sensitive to DNA supercoiling [27–29], a feature that it shares with the Int tyrosine integrase recombinase of bacteriophage λ [30, 31]. Inhibition of type II topoisomerase activity by the drug novobiocin results in a dose-dependent relaxation of negatively supercoiled DNA and concomitant biasing of *fimS* inversion in favour of the ON orientation [27]. In λ, DNA relaxation favours excision of the prophage from the chromosome while negative DNA supercoiling is required for efficient λ integration [30, 31]. Thus, in both of these tyrosine integrase-mediated site-specific recombination systems, DNA topology exerts differential effects on the directionality of the reaction.

Three nucleoid-associated proteins (NAPs) influence the inversion of *fimS*: the integration host factor, IHF [32–34]; the leucine-responsive regulatory protein, Lrp [35–38]; and the histone-like nucleoid structuring protein, H-NS [36, 39–41].

IHF is essential for inversion; in its absence the *fimS* element becomes frozen in either the ON or the OFF orientation, reflecting the switch phase at the moment that IHF was removed from the cell [33, 34]. IHF binds to two sites; the IHF-1 site is adjacent to the left inverted repeat (IRL) of *fimS*, while site IHF-2 is within *fimS* (Fig. 1a). Site IHF-2 has an ancillary role in boosting the activity of the *fimA* promoter [33], while IHF-1 is essential for phase OFF-to-ON orientational bias [36]. IHF acts in concert with Lrp to impose phase ON orientational bias; Lrp binds to two sites, LRP-1 and LRP-2 (Fig. 1a), within *fimS* [37], and its presence is required for phase ON biasing when DNA is relaxed [38]. Once phase ON bias is established, the H-NS NAP is required to maintain it. This is achieved when H-NS binds to *fimS* and to the adjacent chromosomal DNA, creating a nucleoprotein 'trap' that maintains *fimS* in the ON orientation under conditions of relaxed DNA topology [36, 40]. Thus, Lrp acts as a directionality determinant in *fimS* site-specific recombination, by analogy with the role of the Xis protein during bacteriophage λ excision from the *E. coli* chromosome, catalysed by the Int tyrosine integrase [42].

Lrp binds cooperatively to DNA [43] at sites matching a degenerate consensus sequence [44], its 8-mer/16-mer oligomeric structure is sensitive to l-leucine [45] and its gene regulatory activities may be indifferent to, stimulated by, or inhibited by l-leucine and other amino acids [43, 46, 47]. The production of Lrp is subject to transcriptional autorepression [10, 48] has a broad impact on gene expression [49] and plays a central part in the adaptation of the bacterium to the stationary phase of the growth cycle [50–52].

In contrast to Lrp, the factor for inversion stimulation, Fis, is produced predominantly in the early exponential phase of growth [53–56]. It influences a wide range of DNA transactions: site-specific recombination [42, 57]; chromosome replication [58–61]; transcription [62–64]; and transposition [65, 66]. The Fis protein influences DNA topology at several levels: it regulates the transcription of *topA,* the gene that encodes DNA topoisomerase I [67, 68], and also the expression of the *gyrA* and *gyrB* genes, encoding the alpha and beta subunits, respectively, of the heterotetrameric DNA gyrase [69, 70]. In addition, Fis acts as a topological buffer to set local DNA topology by constraining plectonemically supercoiled DNA [71, 72]. Negative DNA supercoiling stimulates the transcription of the negatively autoregulated *fis* gene [73].

Fis is required to maintain the OFF orientation of *fimS* in the presence of the FimE recombinase [74], suggesting that Fis is another directionality determinant affecting *fimS* site-specific recombination in the same direction as Lrp. Here, we explore the role of the Fis protein in FimB-mediated *fimS* inversion by monitoring the inversion preferences of this site-specific recombinase in the presence or absence of Fis and by identifying biochemically, and then disrupting genetically, a binding site for Fis within *fimS* that is essential for determining the inversion preference of FimB. This Fis binding site substantially overlaps LRP-2, one of the Lrp binding sites in *fimS,* a situation that is reminiscent of the overlapping binding sites used by Xis and Fis in the *attR* region of the λ prophage during Int-mediated excision of the bacteriophage from the chromosome [31, 42]. The Fis and Xis proteins bind simultaneously to *attR* [42]; in contrast, we found that Fis and Lrp bind competitively to the LRP-2 site in *fimS*.

## Methods

### Media, growth conditions and genetic techniques

The strains used in these experiments were derivatives of *E. coli* K-12 (Table 1). Strain XL-1 Blue was used for routine molecular biology and the *fimA-lacZ* transcriptional fusion strain VL386, and its derivatives, were used for experiments with the *fimS* genetic switch. The VL386 Δ*fis::kan* knockout mutant was derived by P1*vir*-mediated transduction [75, 76] using a CSH50 *fis::kan* mutant lysate. VL386 *lrp::cml* was also prepared by transduction, using a CSH50 *lrp::cml* lysate. Complementation of the *fis* mutation was carried out using plasmid pFIS349, which is a single-copy plasmid based on the mini-F origin plasmid pZC320 [77]. Bacteria were cultured in lysogeny broth (LB, made from Difco media components) or LB agar (containing agar at 1.5 % w/v) [75]. MacConkey lactose agar plates [75] were used for Lac phenotype determination. Unless otherwise stated, liquid cultures were grown overnight at 37 °C with aeration at 200 r.p.m in an orbital incubator (New Brunswick). Where appropriate, antibiotics (Sigma-Aldrich) were used at the following concentrations: carbenicillin (100 μg ml^−1^), chloramphenicol (25 μg ml^−1^) and kanamycin (20 μg ml^−1^). Plasmid DNA was introduced to bacterial cells by CaCl_2_ transformation [78] or electroporation using a Bio-Rad Gene Pulser as described in Hanahan, 1983 [79].

**Table 1.** Bacterial strains and plasmids

Strain/plasmid	Relevant details	Reference/source
**Strains**		
VL386	φ(*fimA-lacZ*)λpL(209)*fimE*::IS*1*	[9]
VL386*recD*	VL386 *recD*::Tn*10*	[24]
CJD2116	VL386 Δ*fis*::*kan*	This work
CJD2117	VL386 Δ*lrp*::*cml*	This work
CJD2119	CJD2116 (pFIS349)	This work
VL386*fimS-dist*	VL386 with Fis/LRP-2 binding site disrupted	This work
XL-1 Blue	*recA1 endA1 gyrA96 thi-1 hsdR17 supE44 relA1 lac* [F' *proAB lacIqZ* M15 Tn*10* (Tet^r^)]	Stratagene
**Plasmids**		
pSGS501	*fimB*::*kan*, *fimE*::IS*1*, φ(*fimA-lacZ*) cloned in pACYC184, switch phase OFF Cm^r^	[24]
pFIS349	pGS349 containing the *Salmonella enterica* serovar Typhimurium *dusB-fis* operon, Ap^r^	[77]
pLSB124	pMAK705 containing wild-type *fimB* and *sacB* gene from *Bacillus subtilis *	This work
pMAK705	Temperature-sensitive allele exchange vector	[109]
pMCL210	Cloning vector, p15A replicon	[110]
pMMC106	pMCL210 cut with NheI and XbaI and religated to delete the *lac* promoter	[20]
pMMC108	*fimS* cloned as a 550 bp fragment in the PstI site of pMMC106	[20]
pSLD203	*fimB* gene cloned in pUC18	[28]
pUC18	ColE1 replicon, Ap^r^	[111]

### Molecular biological techniques

Plasmid DNA was isolated using Qiagen Midi columns or Wizard mini prep columns (Promega). Specific DNA fragments were isolated using an agarose gel extraction kit (Roche Applied Science). Restriction enzyme digests were carried out using enzymes purchased from New England Biolabs by following the manufacturer’s recommended procedure. Plasmid DNA was sequenced using a T7 sequencing system (USB). Plasmid pSGS501 was used as the template with oligonucleotide COL69 as the sequencing primer (Table 2). Automated sequencing was carried out at MWG Biotech. This company also synthesized the oligonucleotides used in this study.

**Table 2.** Oligonucleotides

Name	Sequence	Purpose
OL4	5′-GACAGAACAACGATTGCCAG-3′	PCR switch assay
OL20	5′-CCGTAACGCAGACTCATCCTC-3′	PCR switch assay
BSFORBIO	5′-CCACCTCATGCAATATAAAC-3′	Probe for gel retardation assays
BSREVBIO	5′-CCCCCAAAAGATGAAACATTT-3′	Probe for gel retardation assays
SpvR11	5′-CCAAGCTTCAGTACTGATCTTGCGATACTG-3′	Probe for gel retardation assays
SpvR14	5′-CCCAAGCTTCAGGTCACCGCCATCCTGTTTTTGC-3′	Probe for gel retardation assays
COL-DFP	5′-GAGAAGAGGTTTGATTTAAC-3′	Probe for DNase I footprinting
COL69	5′-GAGTTTGACTGCCAACACT-3′	Probe for DNase I footprinting and primer for Sanger sequencing
FBSFOR2	5′-CAATAGAATATTAAGGGGTTAGCTAAACT- GAAAAAG-3′	Mutagenesis of Fis binding site
FBSREV2	5′-CTTTTTCAGTTTAGCTAACCCCTTAATATTCTATTG-3′	Mutagenesis of Fis binding site

### Inhibition of DNA gyrase with novobiocin

Assays involving the DNA gyrase inhibitor novobiocin (Sigma-Aldrich) were performed as follows: bacteria containing the *fimA-lacZ* transcriptional fusion were screened for their Lac phenotypes on MacConkey lactose indicator medium as described previously [27]. Distinctly phase ON (red/Lac^+^) or phase OFF (white/Lac^−^) colonies were used to inoculate 2 ml LB (lysogeny broth) in test tubes and grown overnight. These were used to inoculate 250 ml flasks containing 25 ml of LB. These cultures were grown aerobically at 200 r.p.m. until they reached an optical density of approximately 0.1 at 600 nm. At this point novobiocin (aqueous stock solution 100 mg ml^−1^) was added to a final concentration of 0, 12.5, 25, 50, 75, or 100 μg ml^−1^. Cultures were incubated for a further 20 h before sampling to determine the orientation of the *fimS* switch in the chromosome.

### Determination of *fimS* orientation on the chromosome

A PCR-based assay was used to determine the orientation of the *fimS* genetic switch on the *E. coli* chromosome (Fig. 1a). This method exploited the presence of a unique BstUI restriction site in the *fim* switch, *fimS,* which results in products of unequal lengths, depending on the switch orientation [24]. This restriction fragment length dimorphism allowed ON and OFF switches to be distinguished and quantified. Bacterial samples were harvested by boiling 50 µl of culture following overnight incubation at 37 °C. Oligonucleotides OL4 and OL20 (Table 2) were used to amplify the switch region and generate a 726 bp DNA product. DNA amplification used *Taq* polymerase (New England Biolabs) with the following PCR conditions: denature at 94 °C for 3 min, followed by 30 cycles of 94 °C for 1 min, 58 °C for 1 min and 72 °C for 1 min. This was followed by a final extension time of 10 min at 72 °C. Samples were cooled to 60 °C, 10 units of BstUI were added to each reaction and incubation was continued at 60 °C for 3 h. Digested PCR products were electrophoresed on 2 % agarose gels. Phase OFF populations of bacteria yielded two DNA fragments of 539 and 187 bp in length, whereas phase ON populations gave fragments of 433 and 293 bp. The well-resolved 539 and 433 bp DNA fragments were used to compute the relative quantities of ON and OFF switches in the bacterial population: QUANTITY ONE image analysis software was used to measure approximate proportions of the resultant fragments (Fig. 1b). The PCR-based DNA inversion assays were performed in triplicate and typical data are shown.

### Analysing protein binding to DNA by electrophoretic mobility shift assay

The association of purified Fis or Lrp proteins with the *E. coli fim* switch was measured using an electrophoretic mobility shift assay (EMSA). A 135 bp probe was amplified by PCR with Pfu polymerase (Stratagene), using the primer pair BSFORBIO and BSREVBIO (Table 2). The *S. enterica spvR* promoter was amplified as a 157 bp fragment using the primer pair, spvR11 and spvR14 (Table 2) [80], and this was used as a negative control for the Fis binding experiments [63]. The probes were then purified using a PCR clean-up kit (Roche Applied Science). The oligonucleotides had been ordered with 5′ biotinylated ends allowing for subsequent complex detection. Complexes were formed following incubation of amplified probe with increasing concentrations (0–270 nM) of purified His-tagged Fis [69] or 0–220 nM of purified His-tagged Lrp [38] for 15 min as described by the manufacturers of the Electrophoretic Mobility Shift Assay kit (Pierce). Competitive binding of purified Fis and Lrp was tested with Lrp being added in increasing concentrations to DNA that had been prebound with Fis at a constant concentration. Protein–DNA complexes were resolved by electrophoresis through a 7.5% polyacrylamide gel for 2 h at room temperature. The gel was then electrophoretically blotted and developed using the procedure recommended by the manufacturer (Pierce).

### DNase I footprinting

A 385 bp fragment encompassing *fimS* was amplified from pSGS501 with the primers COL69 and COL-DFP (Table 2). The PCR product was purified with a PCR clean-up kit (Roche Applied Science) and end-labelled with [γ-^32^P]-ATP (Perkin Elmer) using T4 polynucleotide kinase (New England Biolabs). This fragment was then digested for 2 h with MfeI at 37 °C in a reaction volume of 60 µl. The probe was purified by extraction from a 6% polyacrylamide gel, following electrophoresis in TBE buffer. Labelled DNA was eluted in 3 ml of elution buffer [10 mM Tris–HCl pH 8.0, 1 mM EDTA, 300 mM sodium acetate (pH 5.2), 0.2% SDS] at 37 °C for 48 h. The eluted probe was extracted with an equal volume of phenol : chloroform and ethanol precipitated. The DNA pellet was then resuspended in 100 µl of double-distilled water. Two microlitres of labelled probe solution were used in each footprinting experiment. DNA–protein complexes were formed in 50 µl of footprinting buffer (20 mM Tris–HCl pH 7.5, 80 mM NaCl, 1 mM EDTA, 100 µg ml^−1^ BSA, 10% glycerol and 1 mM DTT) at 37 °C for 30 min. Then 50 µl of 10 mM MgCl_2_–5 mM CaCl_2_ were added and incubation continued for a further 10 min. Next, 0.01 U of DNase I (Roche Molecular Biochemicals) was added, and digestion was allowed to proceed for 1 min. The reaction was terminated by the addition of 90 µl of stop solution (200 mM NaCl, 30 mM EDTA pH 8.0, 1% SDS, 100 µg ml^−1^ tRNA). Samples were extracted once with an equal volume of phenol : chloroform and then precipitated with ethanol and resuspended in 6 µl of gel loading dye. Samples were denatured at 95 °C for 3 min and were subjected to electrophoresis on a 7% urea–polyacrylamide gel alongside DNA sequencing reactions. Dideoxy chain terminator sequencing [81], primed by oligonucleotide COL69 (Table 2), was used to generate the DNA sequence ladder.

### Site-directed mutagenesis and allele replacement

Site-directed mutagenesis was performed using the Quikchange II (Stratagene) site-directed mutagenesis kit, according to the manufacturer’s recommendations. The oligonucleotides used to mutate the Fis binding site (FBSFOR2 and FBSREV2) are described in Table 2, and were supplied by MWG Biotech. Plasmid pMMC108 [20] was used as the substrate for the mutagenesis. The method of allele replacement was as described previously [24]. Briefly, the mutated Fis binding site was introduced to the chromosome by cloning an MfeI-SnaBI fragment of *fimS,* containing the disrupted site into pSGS501, a plasmid containing the *cat* chloramphenicol resistance gene (Table 1). The resulting plasmid was digested with EcoRV and an 8 kb fragment containing the mutated *fimS* region was gel extracted. Two micrograms of this fragment were electroporated into strain VL386*recD*. Loss of plasmid sequences following homologous recombination with the chromosome was confirmed by testing the transformants for chloramphenicol sensitivity. The presence of the disrupted Fis binding site in the chromosomal *fimS* element was confirmed by PCR amplification followed by DNA sequencing.

## Results

### Loss of Fis alters the pattern of *fimS* inversion when DNA gyrase is inhibited

Inversion of *fimS* by the FimB recombinase becomes biased towards the ON orientation when the introduction of negative supercoils by DNA gyrase is inhibited by novobiocin. Increasing the dose of the gyrase-inhibiting drug exacerbates this effect. The Fis protein is known to preserve negative supercoils at a local level by binding to DNA [71, 72]. We investigated the impact of eliminating Fis protein production on the inversion of *fimS* by FimB, using as the wild-type *E. coli* strain VL386, and CJD2116, an isogenic *fis* knockout mutant (Table 1), treated with increasing concentrations of novobiocin (Fig. 1b).

In the wild-type, treatment with incrementally increasing concentrations of novobiocin was accompanied by a progressive accumulation in the bacterial population of *fimS* switches in the ON orientation, in agreement with previous data [27–29, 36, 38]. In the *fis* mutant, this effect was not observed: as the concentration of the drug increased, the switch orientation remained close to a constant ratio of 30% ON and 70% OFF (Fig. 1b). The introduction of a functional copy of *fis* on a single-copy plasmid (strain CJD2119, Table 1) restored the biased OFF-to-ON switching that is characteristic of the wild-type (Fig. 1b). These data implicated Fis as a contributing factor in biasing FimB-mediated *fimS* switching when DNA gyrase activity is inhibited by novobiocin. We decided to investigate the relationship between Fis and *fimS* in more detail at the molecular level.

### Characterization of a Fis binding site within *fimS*

**Fig. 2.**
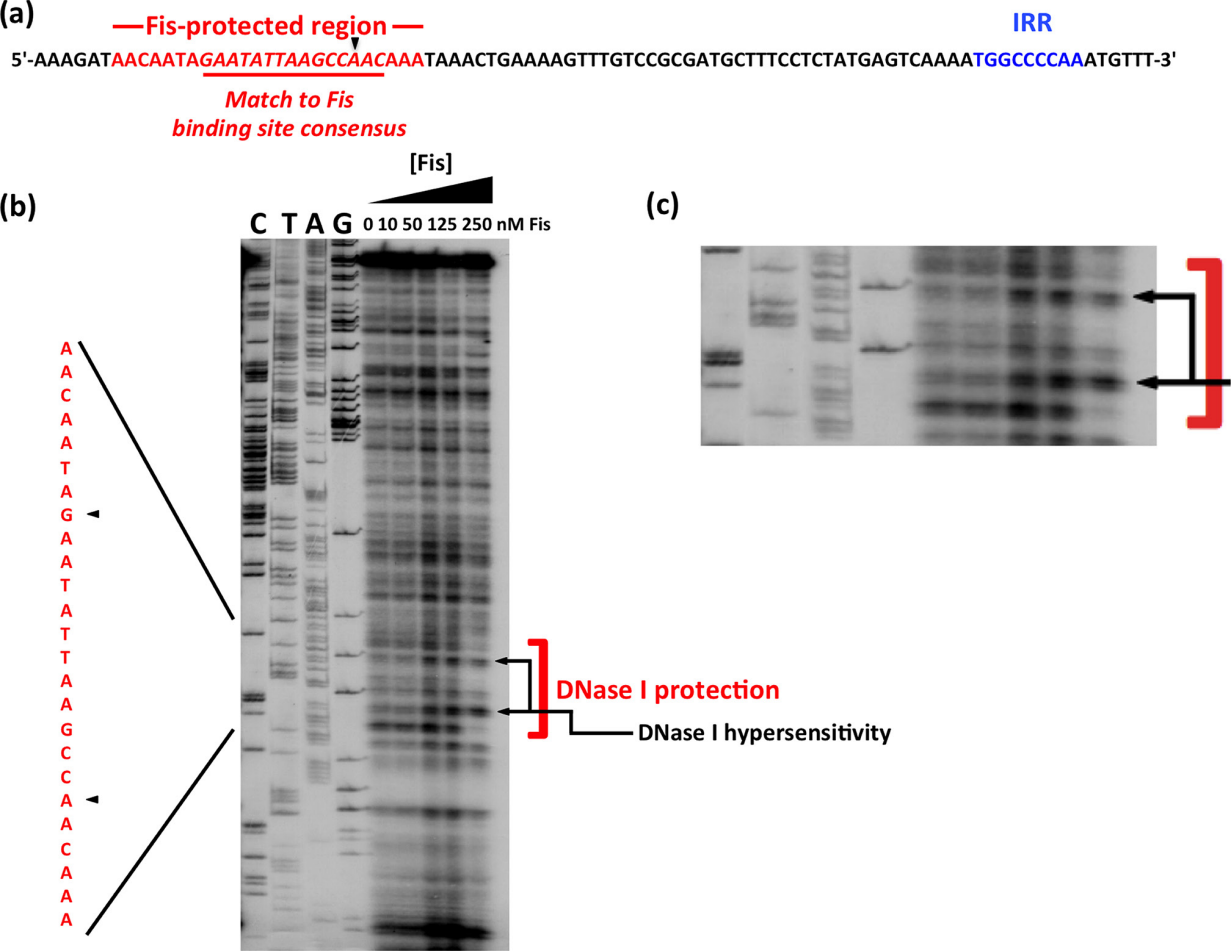
Identifying a binding site for Fis within *fimS*. (**a**) The DNA sequence of the right end of *fimS* in phase OFF, showing the location of the right inverted repeat, IRR (blue), and a sequence matching the consensus for Fis binding sites (red). (**b**) DNase I footprinting was performed with purified Fis protein and a DNA fragment corresponding to the right end of phase OFF *fimS*. Concentrations of Fis are given above each lane. The products of dideoxy chain-terminator nucleotide sequencing reactions, carried out with the same DNA fragment, are shown in the lanes labelled C, T, A and G. The assay revealed a region in which Fis protected the DNA from DNase I digestion, with two nucleotides exhibiting hypersensitivity to the enzyme. The protected region is highlighted with a red bracket and black arrows indicate the two hypersensitive bases. Black arrowheads point to the regions of hypersensitivity in the sequence shown on the left in red; this sequence corresponds to that shown in red in (**a**). This part of the image is reproduced in an enlarged format in (**c**).

Although the consensus sequence of the Fis binding site in DNA is degenerate, it has a number of features that are highly conserved among high-affinity sites [82, 83]. These allowed us to identify, by inspection, a potential binding site for Fis within the *fimS* element, located 50 bp from the 9 bp inverted repeat that forms the right-hand boundary of the switch when in the OFF orientation (Fig. 2a). We then used DNase I footprinting to map the binding site of purified *E. coli* Fis protein on the *fimS* genetic element *in vitro*. The site that was protected by Fis from DNase I digestion corresponded to the DNA sequence that matched with the consensus for high-affinity Fis binding sites (Fig. 2b, c). The DNase I footprint consisted of bases that were protected by Fis and bases that became hypersensitive to digestion in the presence of the NAP. The latter are commonly found in sites occupied by DNA-binding proteins that bend DNA, a known property of Fis [58, 82–84].

**Fig. 3.**
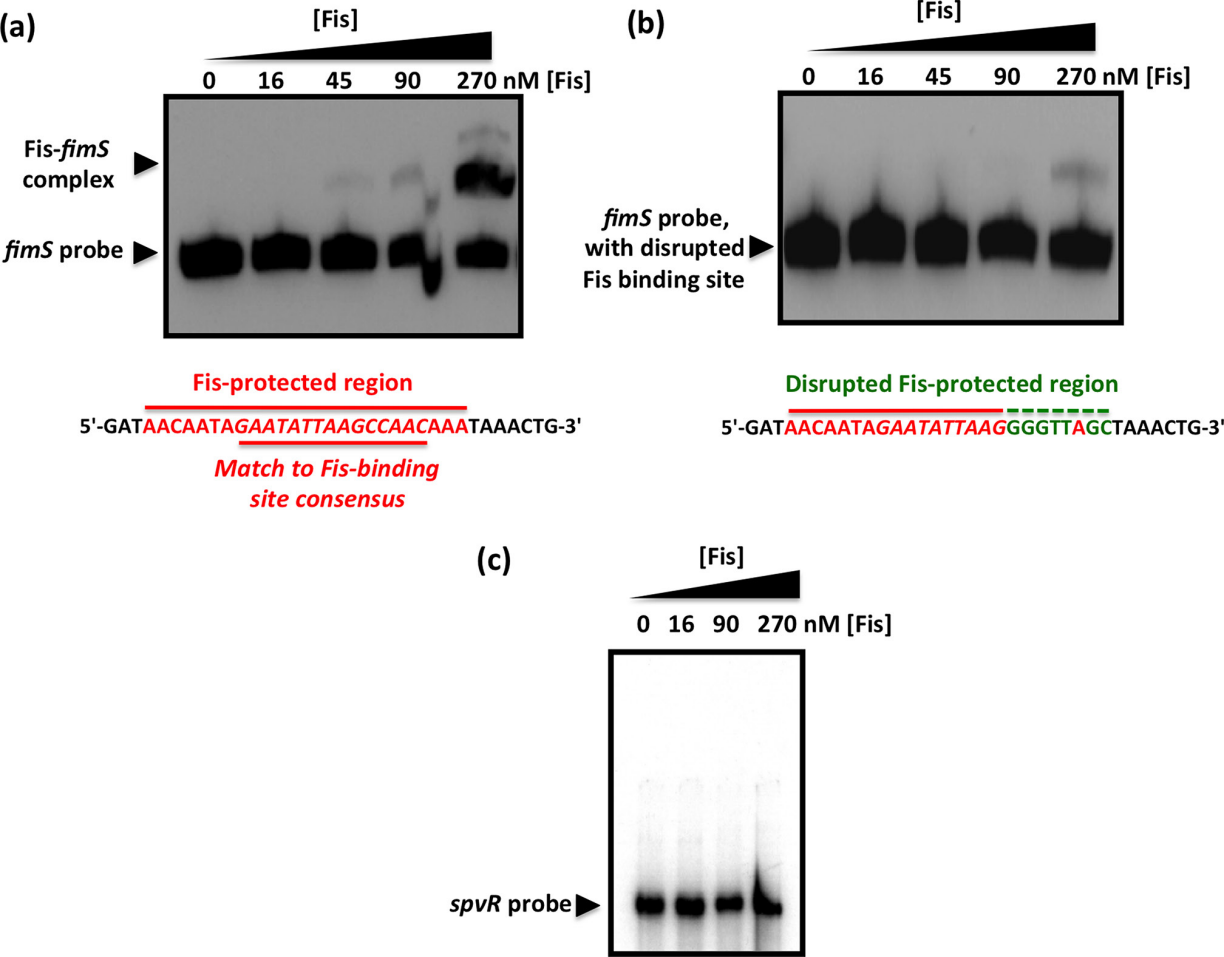
Mutation of the Fis binding site abrogates the Fis-mediated electrophoretic mobility shift of *fimS* DNA. (**a**) EMSA showing the gel mobility shift of a labelled 135 bp fragment of *fimS* DNA that includes the Fis binding site, highlighted in red below the gel. (**b**) Disruption of the Fis binding site by substituting the bases, shown in green below the gel, of the wild-type binding site sequence (red) almost completely abrogated the ability of Fis to alter the electrophoretic mobility of this DNA fragment. (**c**) A control EMSA using a 157 bp DNA fragment corresponding to the *spvR* promoter region from *S*. Typhimurium. This DNA fragment is known not to bind Fis [63]. Purified Fis failed to alter the electrophoretic mobility of the *spvR* DNA fragment at the same protein concentrations used in (**a**). The concentrations of purified Fis used in the experiments are given above each gel lane; black arrowheads indicate the bands corresponding to the unbound DNA probes and in (**a**), the Fis*–fimS* complex.

Further evidence of Fis binding to *fimS* came from an electrophoretic mobility shift assay. At 90 nM Fis, the protein formed a complex that was consistent with the occupation of a single binding site (Fig. 3a). The Fis binding site within *fimS* was subjected to base substitution mutagenesis to alter its DNA sequence without altering its length. Changes were made to eight contiguous bases, destroying the match to the consensus sequence for high-affinity Fis binding sites. The modified DNA element could not bind Fis at a protein concentration of 90 nM, and only a weak interaction was detected at 270 nM (Fig. 3b) that was likely due to the known tolerance of Fis for mismatches to its binding site consensus sequence [82]. Taken together, the DNase I footprinting data and the EMSA results show that Fis binds to the *fimS* genetic switch at a site that is located 50 bp from the inverted repeat boundary at the *fimA* promoter-distal end of *fimS*. The *spvR* promoter region from *Salmonella enterica* serovar Typhimurium, that does not bind Fis [63], was used as a negative control. This DNA sequence did not form a complex with Fis, at this or a higher concentration of the protein (Fig. 3c).

### The Fis binding site is crucial for *fimS* inversion preferences

**Fig. 4.**
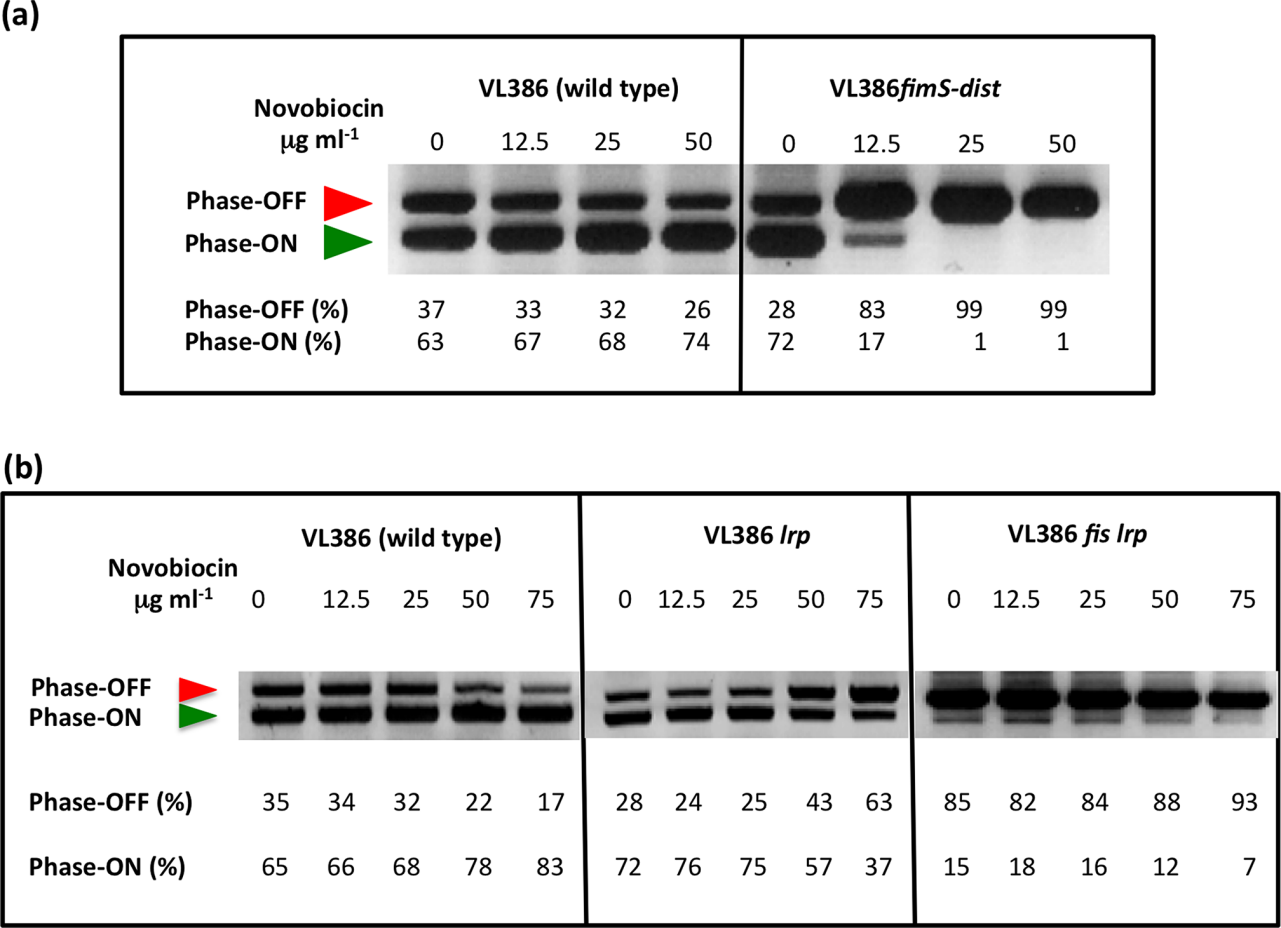
Loss of Fis binding and loss of Fis production biases *fimS* towards phase OFF. (**a**) Electrophoresis of the *fimS* DNA fragments from the wild-type strain (VL386) and from its derivative (VL386*fimS-dist*) in which the Fis binding site in *fimS* is disrupted, following BstUI digestion of the PCR-amplified *fimS* genetic element. (**b**) Electrophoresis of the *fimS* DNA fragments from the wild-type strain (VL386), its *lrp* knockout mutant derivative and the derivative with knockout mutations in both the *lrp* and *fis* genes, following BstUI digestion of the PCR-amplified *fimS* genetic element. The red arrowhead indicates the 539 bp phase OFF diagnostic band and the green arrowhead shows the 433 bp ON diagnostic band. In (**a**) and (**b**), the cultures have been treated with novobiocin at the concentrations given above each gel lane. The intensities of the DNA bands in each lane corresponding to the ON and OFF orientations of *fimS* in each lane were determined by densitometry and are reported as percentages below the lane. The experiment was performed three times and typical data are presented.

The derivative of *fimS* with the 8 bp substitution mutation in the Fis binding site was transferred to the *E. coli* chromosome by homologous recombination. The mutant strain and the wild-type were treated with increasing concentrations of novobiocin and the orientation of *fimS* was monitored by PCR (Fig. 4a). In the wild-type, the *fimS* element adopted a dose-dependent preference for the phase ON orientation with novobiocin treatment; in the mutant with the disrupted Fis binding site, *fimS* adopted a novobiocin-dependent preference for the OFF orientation, the opposite to the situation seen in the wild-type (Fig. 4a). Not only had the direction of the inversion bias been reversed compared to the wild-type, the response to novobiocin occurred at the lowest concentration of the drug (12.5 μg ml^−1^). These results demonstrated that the Fis binding site plays a pivotal role in determining both the direction of the DNA inversion response and the sensitivity of *fimS* inversion to DNA gyrase inhibition. Inspection of the Fis binding site’s location suggested that it overlapped Lrp binding site LRP-2 (see below) and the base substitution mutations might also have impaired Lrp binding to that site. For this reason, the strains used in the *fimS* orientation assay contained a plasmid pUC18 derivative, pSLD203, over-expressing the FimB recombinase (Table 1). This is an established way to allow *fimS* inversion to continue in strains deficient in co-factor production/binding without affecting the response of *fimS* recombination to DNA relaxation [24, 28, 36, 38].

### The *fimS* Fis binding site substantially overlaps a binding site for Lrp

The *fimS* DNA sequence that is protected from DNase I digestion by Fis overlaps the previously characterized LRP-2 binding site used by the leucine-responsive regulatory protein, Lrp. This Lrp site helps to determine the inversion bias of *fimS* [36, 38]. We first studied the *fimS* inversion pattern in *lrp* and *lrp fis* knockout mutants with increasing concentrations of novobiocin, compared with the wild-type pattern (Fig. 4b). The wild-type culture followed the usual pattern, with *fimS* becoming progressively biased towards the phase ON orientation as the novobiocin concentration increased. The *lrp* mutant (CJD2117, Table 1) became mildly biased towards phase OFF, in agreement with previous findings; full phase OFF biasing requires the disruption of both LRP-1 and LRP-2 [38]. In the *lrp fis* double mutant, the switch was already biased towards phase OFF before drug treatment and became almost wholly phase OFF as novobiocin was introduced (Fig. 4b).

**Fig. 5.**
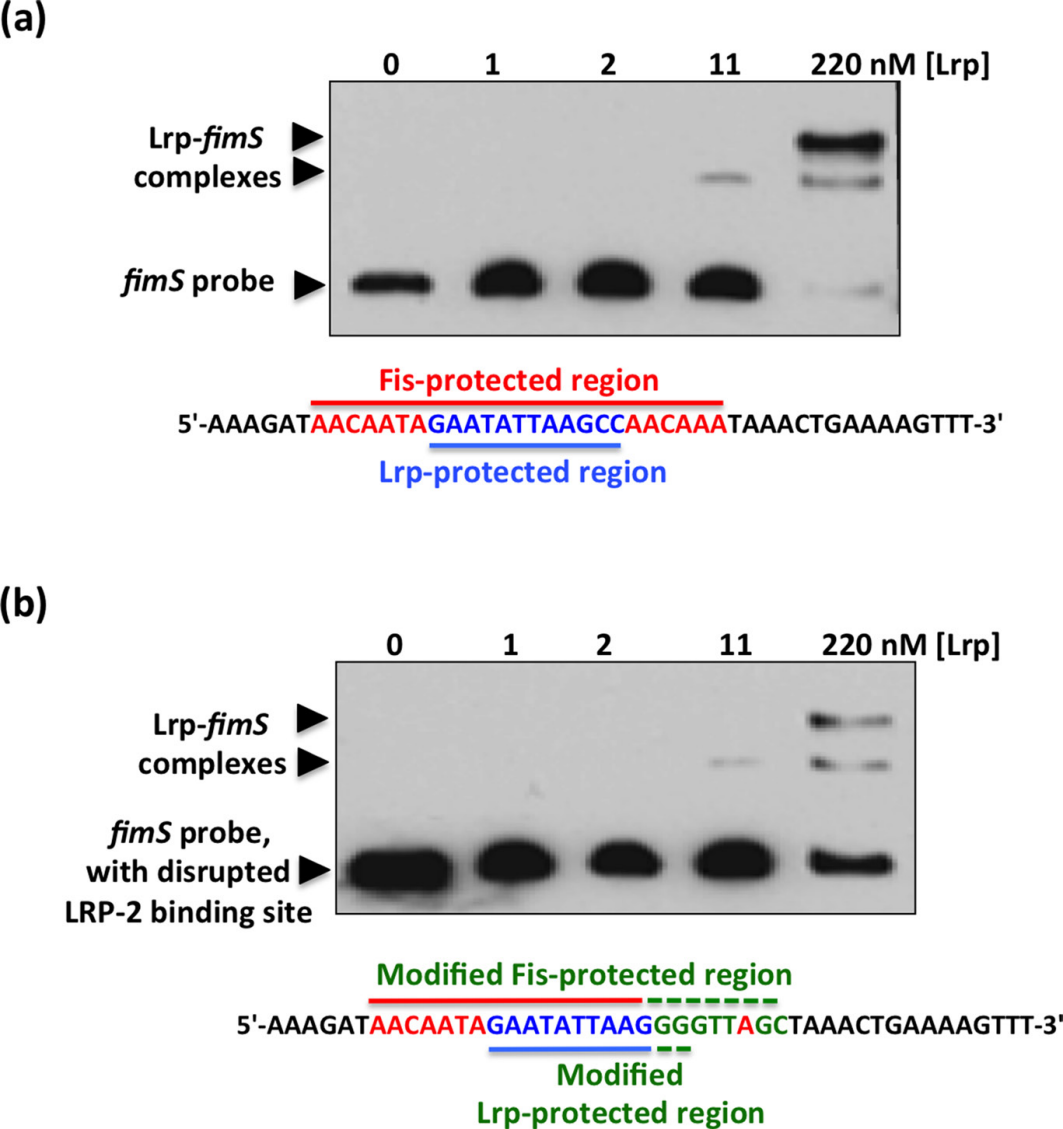
EMSA showing Lrp binding to *fimS* with an intact or a disrupted Fis binding site. (**a**) Purified Lrp protein was incubated at the concentrations shown with a PCR-generated 135 bp DNA fragment from *fimS* that contained an intact Fis binding site. The sequence of the region protected from DNase I digestion by Fis is shown in red and the Lrp binding site (LRP-2) is shown in blue. (**b**) The EMSA was repeated using the *fimS* derivative with the disrupted Fis binding site. The base substitutions are shown below the gel in green, together with the unchanged bases from the Fis binding site (red) and the LRP-2 site (blue). In both (**a**) and (**b**), arrowheads show the positions of bands corresponding to the unbound DNA probe and the Lrp*–fimS* complexes.

We next investigated the effect of the base substitutions that abrogated Fis binding to *fimS* on the binding of Lrp to the invertible switch. These sequence changes had only affected 2 bp of the Lrp-protected region at the LRP-2 site (Fig. 5). The 135 bp *fimS* probe used in the Fis EMSA contains both the LRP-1 and LRP-2 sites. Binding of Lrp to *fimS* produces a number of complexes that depend on the occupancy of the LRP-1 and LRP-2 sites, individually and collectively [37, 38]. Data from EMSA experiments using the *fimS* probe, with and without the Fis binding site mutation, showed that formation of the most electrophoretically retarded *fimS*–Lrp complex was reduced at the highest concentration of purified Lrp (Fig. 5). These data were consistent with the previously described effect of disrupting the LRP-2 site on LRP–DNA complex formation at *fimS* [38] and with the LRP-2 site also being targeted by the Fis protein.

### Lrp displaces Fis from the *fimS* genetic switch

**Fig. 6.**
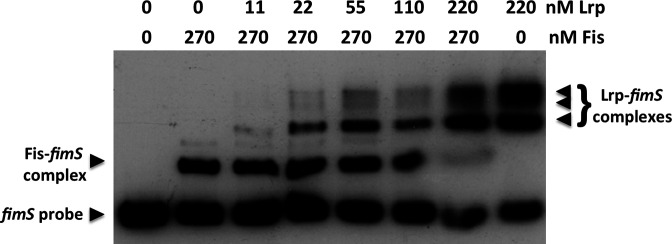
Displacement of Fis from *fimS* by the Lrp protein. Either 0 or 270 nM of purified Fis protein was prebound to a 135 bp fragment of *fimS* DNA containing the LRP-1, LRP-2 binding sites and the Fis binding site. Increasing concentrations of purified Lrp protein were added to the Fis*–fimS* complex and resolved by electrophoresis. Control lanes containing *fimS* DNA with no protein, with just Fis, or with just Lrp were included as controls. Arrowheads indicate the unbound *fimS* probe, the Fis*–fimS* complex and the Lrp*–fimS* complexes. At 220 nM, Lrp almost completely displaced prebound Fis from *fimS*.

**Fig. 7.**
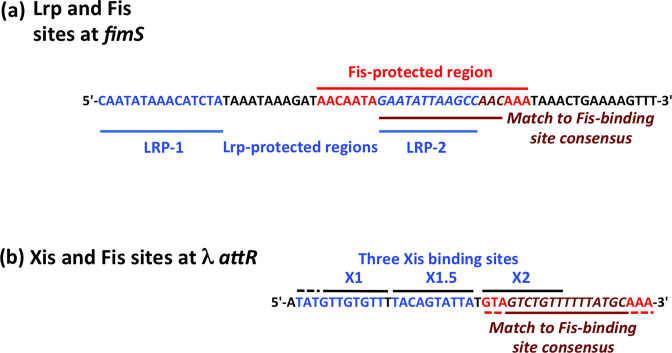
The Fis binding regions in *fimS* and in the bacteriophage λ prophage. (**a**) A summary of the DNA sequences of the LRP-1 and LRP-2 binding sites (blue) in *fimS*, showing that the LRP-2 (blue italics) site is nested completely within the Fis binding site (red/crimson). (**b**) The three binding sites for the λ Xis excisionase, X1, X1.5 and X2 (blue) are shown. The Fis site (red/crimson) overlaps the X2 site completely. Despite the superficial similarity of the nested relationships of the LRP-2 and Fis sites in *fimS,* and the X2 and Fis sites in λ, the interactions between the proteins in each system differ: in *fimS,* Fis competes with Lrp for access to the Fis binding site, whereas Fis and Xis bind cooperatively to the phage DNA in the λ prophage.

The data obtained thus far show that the Lrp and Fis proteins both target the LRP-2 binding site in *fimS* (Fig. 4a, b). Fis is available in high concentration at the onset of exponential growth, before becoming rapidly diluted by cell division as the bacteria in the culture expand in numbers. Since Fis and Lrp both influence *fimS* inversion in the same direction, we hypothesized that Fis might be replaced by Lrp at the LRP-2 site when Fis concentration declines as the exponential phase progresses. It is also possible that Lrp might actively displace Fis through competition for the same binding site. Therefore, we next assessed the ability of Lrp to displace Fis from LRP-2 in a competitive EMSA. Here, the *fimS* DNA was preloaded with purified Fis at a constant concentration and purified Lrp was added at increasing concentrations (Fig. 6). Lrp and Fis complexes with *fimS* could co-exist at intermediate concentrations of Lrp (22 to 110 nM), presumably indicating occupation of the LRP-1 site by Lrp and of LRP-2 site by Fis, but at the highest concentrations of Lrp, the Fis*–fimS* complex was only weakly detected, presumably because the LRP-2 site was now occupied by Lrp on most *fimS* copies in the reaction (Fig. 6). The EMSA competition showed a specific Fis*–fimS* complex and Lrp*–fimS* complexes; we did not detect evidence of a Fis-plus-Lrp complex with *fimS,* in which Lrp occupied both the LRP-1 and LRP-2 sites with co-binding of Fis and Lrp to LRP-2. Thus, the binding pattern of Fis and Lrp at *fimS* differed from the pattern seen with Fis and Xis at the λ *attR* site, where both Fis and Xis act as directionality determinants in λ excision from the chromosome and both bind to overlapping sites in the DNA simultaneously (Fig. 7) [31, 42, 85].

## Discussion

Our data reveal a delicate interplay between DNA supercoiling/relaxation, Lrp and Fis in determining the directionality of the FimB-mediated site-specific recombination reaction in *E. coli *. The *fimS* switch (Fig. 1a) becomes progressively biased towards the ON orientation following novobiocin-induced inhibition of DNA gyrase, the topoisomerase that introduces negative supercoils into DNA (Fig. 1b) [27–29, 36, 38]. In an *lrp* knockout mutant, DNA relaxation results in a reversal of *fimS* inversion outcomes in favour of the OFF, rather than the ON, orientation [36, 38]. Inactivation of Fis production in the *lrp* knockout mutant produces an even stronger preference for the ON orientation, one that is achieved even in the absence of novobiocin, but which becomes much more pronounced as concentrations of the drug increase (Fig. 4). The relationship between Fis and Lrp at *fimS* has similarities to the relationship between Fis and the Xis directionality determinant at *attR* in bacteriophage λ excision (Fig. 7).

Int-mediated excisive recombination of bacteriophage λ is enhanced when DNA supercoiling levels are low, whereas integrative recombination requires negative supercoiling of the phage DNA [30, 85]. The phage-encoded Xis architectural protein stimulates excision by a factor of 10^6^ while simultaneously inhibiting reintegration of the phage [86, 87]. Xis binds to the X1, X1.5 and X2 sites in the *attR* arm of the λ prophage to form a microfilament [88], with site X2 overlapping a binding site for Fis, the F site [89] (Fig. 7). Initially it was thought that Fis substituted for Xis at site X2, allowing excision to proceed under conditions where Xis was limiting [31]. It is now understood that Xis occupies all three of its binding sites, with Fis binding simultaneously to its F site (Fig. 7), producing a nucleoprotein complex with a DNA conformation that is optimal for excisive recombination [42, 88, 90]. The role Fis plays in recruiting Xis does not seem to involve protein–protein contact, but is achieved through DNA allostery [91]. While Xis imposes a preference for excision on the λ prophage, the Fis protein has been reported to stimulate integration as well as excision [57], especially in the absence of Xis [92].

Thus, the λ excision complex differs from the *fimS* OFF to ON inversion complex in that Xis and Fis bind together to overlapping sites in *attR,* while Lrp and Fis bind competitively to overlapping sites in *fimS* (Fig. 6). Despite this distinction, the two systems share a preference for relaxed DNA to facilitate a direction-specific recombination reaction; a dependence on tyrosine integrase recombinases to catalyse the reaction; and a requirement for IHF to occupy two sites in the DNA to organize a recombination substrate with an appropriate architecture (Figs 1a and 7).

Int differs from the FimB and FimE recombinases in that it makes contact with the DNA through both its amino-terminal and its carboxyl-terminal domains (NTD and CTD, respectively) at up to nine sites [31]. The smaller fimbrial recombinases lack the corresponding NTD and only make contact with *fimS* at four sites, two flanking each of the 9 bp inverted repeats, IRL and IRR, that form the boundaries of *fimS* [20, 23]. DNA cleavage and ligation take place within these inverted *fimS* repeats [20] while Int cleaves and religates the λ prophage within the 7 bp inverted repeats that are flanked by Int-CTD binding sites in *attL* and *attR* [31]. In λ site-specific recombination, Fis can stimulate both integration and excision; in *fimS,* Fis plays a role as a directionality co-determinant with Lrp, favouring the ON-to-OFF reaction.

DNA relaxation is a feature of stationary phase cultures [93, 94] and is a condition that biases *fimS* towards the ON phase [27–29, 36, 38]. This bias requires both Fis and Lrp (Fig. 4b). Loss of either protein introduces an alternative bias towards the OFF phase as DNA relaxes [36, 38]; loss of both proteins results in *fimS* being maintained in the OFF phase in almost all bacteria in the population (Fig. 4b).

The competitive relationship described here for Fis and Lrp at *fimS* is reminiscent of the competition between the Dam methylase and Lrp for access to overlapping sites in the regulatory region of *pap*, the operon that encodes Pap pili in uropathogenic strains of *E. coli* [95, 96]. The *fim and pap* operons engage in regulatory crosstalk via PapB-mediated repression of *fim* operon transcription [97–99]. Although DNA recombination does not contribute to the operation of the phase-variable *pap* switch, the outcome of the Dam/Lrp competition determines whether the *pap* operon will or will not be transcribed. Lrp accumulates in stationary phase cultures growing in rich media [100], while Dam concentrations decline under those same growth conditions [101]. It has been suggested that the shift in the Dam/Lrp balance in favour of Lrp facilitates a shift in Pap production from the ON to the OFF phase [96]. These features of *pap* gene regulation by Dam and Lrp mirror those described here for *fim* gene regulation by Fis and Lrp. Like Dam, Fis is produced in decreasing amounts as stationary phase approaches, while the production of Lrp increases [100]. In the early exponential phase, the abundant Fis protein collaborates with the less abundant Lrp to bias *fimS* towards the OFF phase, expanding the number of afimbriate, planktonic bacteria in the population. As exponential growth gives way to growth stasis, the Fis concentration declines sharply and Lrp replaces it at the LRP-2 site in *fimS*, if necessary by competitive displacement.

Biasing the *fimS* switch towards the OFF orientation when Fis is abundant and DNA is negatively supercoiled links *fim* switch inversion preferences to bacterial physiology. The Fis protein is maximally abundant at the beginning of the exponential phase of growth and is almost undetectable by the onset of stationary phase [53–56]. As Fis levels decline, Lrp is available to replace it at *fimS,* prolonging the bias towards phase OFF as long as the DNA remains negatively supercoiled. However, stationary phase is a period of reduced metabolic flux, producing a reduced [ATP]/[ADP] ratio that is unfavourable for the negative DNA supercoiling activity of DNA gyrase [94, 102–105]. At this stage of the growth cycle, DNA becomes relaxed [93, 94, 106], a condition that biases the *fimS* switch to the ON phase in the presence of Lrp [27–29, 36, 38]. Removal of Lrp from *fimS* reverses the inversion bias back towards the OFF phase [38].

The increased representation of fimbriate bacteria in the population of late-exponential phase/stationary phase cells promotes bacterial attachment to abiotic and biological surfaces, with an associated production of biofilm [29, 107, 108]. The resulting transition from a planktonic to a community-based attached lifestyle within the protective shield of a biofilm enhances the survival chances of the bacterial population during a period of unfavourable environmental conditions. Overall, our findings describe a molecular mechanism by which the bet-hedging strategy represented by the stochastic inversion of *fimS* is suspended in favour of the more deterministic outcome of ensuring that type 1 fimbriae are produced by a majority of bacteria in the population [13]. Fis and Lrp are required, in association with DNA relaxation, for the implementation of this deterministic strategy.
